# Top-down effects of fire salamander larvae (*Salamandra salamandra*) on benthic organisms differs between habitat types

**DOI:** 10.1038/s41598-025-97458-6

**Published:** 2025-04-16

**Authors:** Christoph Ptatscheck, Laura Schulte, Jonathan Berger, Barbara A. Caspers

**Affiliations:** 1 Department of Behavioural Ecology, Bielefeld University, Konsequenz 45, 33615 Bielefeld, Germany; 2 JICE, Joint Institute for Individualisation in a Changing Environment, University of Münster and Bielefeld University, Bielefeld, Germany

**Keywords:** Feeding ecology, Meiofauna, Habitat adaptation, Feeding behavior, Niche conformance, Freshwater ecology, Herpetology, Animal behaviour

## Abstract

Fire salamander larvae are top predators in limnic habitats and feed on a wide spectrum of prey. For our study, we hypothesized that the larvae have a top-down effect on meiofauna, but that this effect varies depending on the habitat the larvae originate from. Therefore, we collected larvae from ponds and streams and placed them individually into microcosms with sediment and benthos. After either one week or two weeks, we removed the larvae and counted the number of nematodes, oligochaetes, and rotifers. Already after week one, the abundance of meiofauna was significantly reduced, as was their biomass. Nematodes were more strongly reduced by pond larvae during this week, while oligochaetes were primarily affected by stream larvae but not by pond larvae, regardless of the sampling time. The rotifers were reduced by larvae from both habitats, but only during the first week and not during the second week. Our findings suggest that fire salamander larvae not only have a top-down effect on pelagic or macrobenthic organisms, as often described in other studies, but can also shape the community of small endobenthic organisms. Depending on habitat-specific adaptations in feeding behavior, morphology, or physiology, these effects can vary.

**Supplementary Information:**

The online version contains supplementary material available at 10.1038/s41598-025-97458-6.

## Introduction

Salamander larvae are top predators in fish-free waters such as temporal ponds or headwater streams^1^. The results of numerous studies on the gut contents indicate that their diet includes invertebrates from the water column as well as benthic taxa such as insect larvae (e.g., Chaoboridae, Culicidae and Chironomidae), crustaceans (e.g., Copepoda, Cladocera and Isopoda) and oligochaetes^2–6^. Fire salamander larvae can occur in different habitats, and the food composition of pond and stream larvae can differ greatly^7^. By exerting top-down pressure, salamander larvae can significantly reduce the abundance of certain planktic and macrobenthic taxa in lentic systems^3,4,8^, which in turn may have indirect effects on other species of the community^9,10^. Furthermore, the ingestion of aquatic insect larvae regulates the number of hatching insects and thus also affects the surrounding terrestrial habitats^4,5,11^.

With increasing larval development and body size, the prey spectrum also increases, which is often explained by the larger mouth size and thus the ability to handle larger prey^2,12,13^. Bell^14^ studied the feeding habits of newt larvae and attributed this change in food intake to a shift from ambush predation to more active hunting behavior during the course of larval development. Although it is assumed that diet composition largely reflects the composition of prey in the habitat^15^ and corresponding seasonal changes^7^, some studies indicate specialized hunting by salamander larvae^12,16,17^, such as a preference for slower prey. However, the trophic impact of salamander larvae on endobenthic organisms, especially meiofauna such as oligochaetes, nematodes or microcrustaceans (benthic organisms that pass through a sieve with 500 μm meshes and are retained by a 20 μm sieve, according to Ptatscheck et al.^18^, is not yet fully understood, and studies from lotic habitats are largely absent (but see ^19^). However, meiofauna are found in high abundances (sometimes > one million ind. m^− 2^) in headwater streams or ponds^20–22^ and represent a standing stock of food resources for larger organisms such as macroinvertebrates or juvenile fish; however, they cannot be reliably detected via gut analysis because of their rapid digestion^23,24^.

Salamander larvae are classified as typical suction feeders, generating unidirectional water inflow by opening their mouths and expanding the buccal cavity to capture single prey organisms at close range from the water column or surface (reviewed by ^15^). The water is then released outwards through the gill slits. However, dietary analysis of salamander larvae’s diet revealed that even deposit feeding and possibly filter feeding processes may be of importance. For example, Donovan and Folkerts^25^ showed that the gut content of *Desmognathus aeneus* comprised 16% detritus. At the same time, high densities of endobenthic meiofauna, such as nematodes or microcrustaceans were found in the gut^6,7,26^, whose uptake cannot be explained by selective suction feeding from the sediment surface or by accidental bycatch alone. Rather, this indicates that sediments could also be ingested directly and that the small prey could be filtered out. In fact, the larvae of several salamander taxa, such as fire salamander (*Salamandra salamandra)* possess gill rakers, spiny protrusions on the gill arches that are commonly associated with the filtration of small prey organisms^15,27,28^. These structures are already present in embryos, and their number on the gill arches and size continue to increase during larval development before being reduced during metamorphosis^27^. Similar morphological structures also enable bottom-biting fish like carps or gudgeons to effectively filter small organisms from the sediment and affect their abundance, biomass and diversity through strong top-down pressure^23,24^.

In our study, we investigate whether fire salamander larvae can exert a general trophic impact on benthic communities, with a focus on the meiofauna. Fire salamander females deposit fully developed larvae in both ponds and headwater streams^29,30^, but also prefer sections with little current in lotic habitats^31^. In contrast to streams, ponds are characterized by periodic desiccation, higher temperatures, lower oxygen levels, higher predation risk (e.g., by newts or water beetles), increased competition and lower prey availability for salamander larvae^32^. As adaptations to these unfavorable living conditions, larvae have a greater birth weight, develop a more pronounced gill system and metamorphose earlier than their relatives do in streams^29,30,33^. Oswald et al.^34^ reported more pronounced shelter-seeking behavior for pond larvae, and Krause et al.^35^ found that larvae reared under poor nutritional conditions to also seek shelter more often than do larvae reared under rich nutritional conditions.

In order to investigate the influence of these habitat-related characteristics on feeding behavior, we performed standardized laboratory experiments in microcosms with both pond and stream larvae. These microcosms were filled with natural sediment containing a whole benthic community, and one salamander larva was added. After one and two weeks, the composition, abundance and biomass of the benthic organisms were examined. The identical starting conditions enabled us to assess and compare the influence of the respective larvae optimally. Following our preregistration^36^, we assumed that (1) fire salamander larvae reduce the abundance and biomass of benthic invertebrates in the experimental microcosms through predation. However, (2) this top-down effect will be stronger in treatments with pond larvae because they are adapted to use the few available food resources more efficiently to be able to leave their temporary habitat as early as possible. Thus, (2a) they will consume even small organisms like nematodes, whereas (2b) their stream relatives will consume mainly larger oligochaetes. Additionally, we expected that (3) pond larvae will be less active while foraging, as they are less risk prone and, again, are able to use food resources more efficiently.

## Methods

### Organisms

The salamander larvae investigated in this experiment originated from populations that colonize headwater streams and temporal ponds (mostly water-filled bomb craters) in the Kottenforst, a forest area southwest of Bonn (Germany, N 50°40’50.6748, E 7°7’23.1204). In the spring of 2023, we collected 25 stream and 23 pond larvae of approximately the same size (3.0–3.5 cm body length) to avoid a size effect and took them to the laboratory for further examination. We collected larvae from two ponds (13 and 10 larvae) and two streams (13 and 12) to avoid any location-specific effects (e.g., sibling effects). We were not able to find more larvae of the same size in the two ponds. Larvae that were physically impaired (e.g., injured limbs or tails) were excluded from this experiment. The larvae were kept individually in microcosms (see below) filled with stream water (from a different location, see below) at a water temperature of 15 °C and a light cycle of 12:12 h for one week to acclimate to the laboratory conditions. During this time, the larvae were not fed to achieve a greater trophic effect during the experiments.

Water, sediment and containing benthic organisms were collected from Johannisbach, a second-order headwater in Bielefeld (Germany). Furthermore, we transferred sediment and water to buckets, mixed and filtered the supernatant through a 10 μm sieve, and added the retained material to the sediment already collected (similar to ^37^). This increases the abundance of benthic organisms. We stored the collected material in a plastic box (60 × 40 cm floor area, 3 cm sediment height), which was covered with stream water, aerated with air stones and acclimatized to the laboratory conditions for one week (see above). We decided not to use water from the larvae’s respective original habitats for the study to minimize mortality of benthic organisms due to alterations in water quality, thereby ensuring reliable and consistent abundances during the experimental period. Since this stream section has been well-documented in previous investigations, the presence of predators such as fish, anisopterans, large predatory coleopterans, or newts at the sampling site can be excluded. Similarly, the chemical cues of these taxa can be ruled out, except for fish, which are present in the upstream sections.

### Study design

**Fig. 1:**
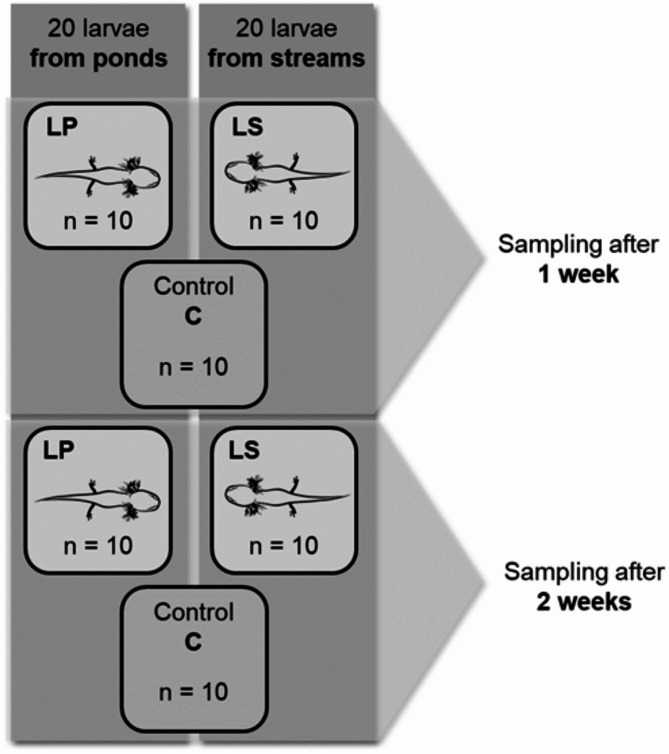
Experimental design. Twenty fire salamander larvae (*Salamandra salamandra*) from each habitat type (pond, stream) were placed into individual microcosms filled with sediment and benthic organisms. After one week, 30 microcosms (10 with larvae from ponds, LP, 10 with larvae from streams, LS, and 10 controls, C) were sampled. After another week, the remaining 30 microcosms were sampled.

Before the start of the experiment, the sediment was thoroughly homogenized in a single box. Then, we filled the Plexiglas microcosms (10 × 10 × 6 cm), each with 1.5 cm of sediment, which in turn was covered with 3 cm of stream water. We placed one pond larva (LP) in each of the 20 microcosms and one stream larva (LS) in each of the other 20 microcosms (Fig. 1). The other larvae (5 from streams and 3 from ponds) served as reserves to compensate for any losses. We randomly assigned the larvae within the treatment groups (LP, LS) to the microcosms. Twenty of the remaining microcosms did not contain larvae and served as control treatments (C). After one week, we sampled 30 microcosms (10 per treatment + 10 controls). Therefore, we placed the larvae, if any, back in their aquarium and transferred the sediment and water to 500 ml PE bottles. The remaining microcosms were treated in the same way after week two (Fig. 1). Once a group of larvae left the experiment, we transferred them back to their original habitat in the Kottenforst. Before and after the experiment, we measured the total body length of each larva and the gill filament length by using a camera and ImageJ. During the experiment, we also recorded the daytime movement of the larvae with four video cameras that were installed above the microcosms (60 cm distance). Due to technical difficulties, we could analyze only the records from the daytime. We measured the average speed of the moving larva, the distance it travelled and the total proportion of time that it was moving within its microcosm via the software AnimalTA^38^ Version 2.3.1.

### Sample processing and counting of organisms

We stained all the samples with Rose Bengal and preserved them in 4% formaldehyde. The benthic organisms were extracted from the sediment by centrifugation (LUDOX TM 50, 1.14 g ml^− 1^, mesh size 10 μm) as described by Pfannkuche and Thiel^39^ and counted in gridded Petri dishes under a Leica S6E stereomicroscope at 45x magnification. This method allowed us to gain knowledge about the abundance of organisms rather than receiving present/absent data through eDNA analysis. Furthermore, we divided all macro- and meiobenthic organisms into taxon-specific size classes to calculate the respective biomass (dry weight) according to Brüchner-Hütteman et al.^40^.

### Statistical analysis

We investigated differences in the benthos abundance, biomass and nematode size classes of the treatments for the first and second week using a Kruskal-Wallis test and, if needed, a Kruskal‒Meier (MC) post hoc test. Similarly, we calculated differences in the biomass in the first and second week. To test for differences between the sampling times, we calculated the percentage change in abundance or biomass and used a Mann‒Whitney U test.

For the analysis of the video data, we used a linear mixed effect model (LMM) with the R packages lme4^41^. First, we used the Shapiro‒Wilk test to test for normality and variance. In case normality of the data is not given, we used the bestNormalize package. We used the proportion of time moving or average speed or distance traveled as the dependent variable, habitat type (pond, stream), size and week (week 1, week 2) as well as the interaction between week and habitat type as fixed factor and ID as random factor. All tests were performed using R (version 4.3.2, 2023-10-31). We set the significance level to α = 0.05.

## Results

**Fig. 2:**
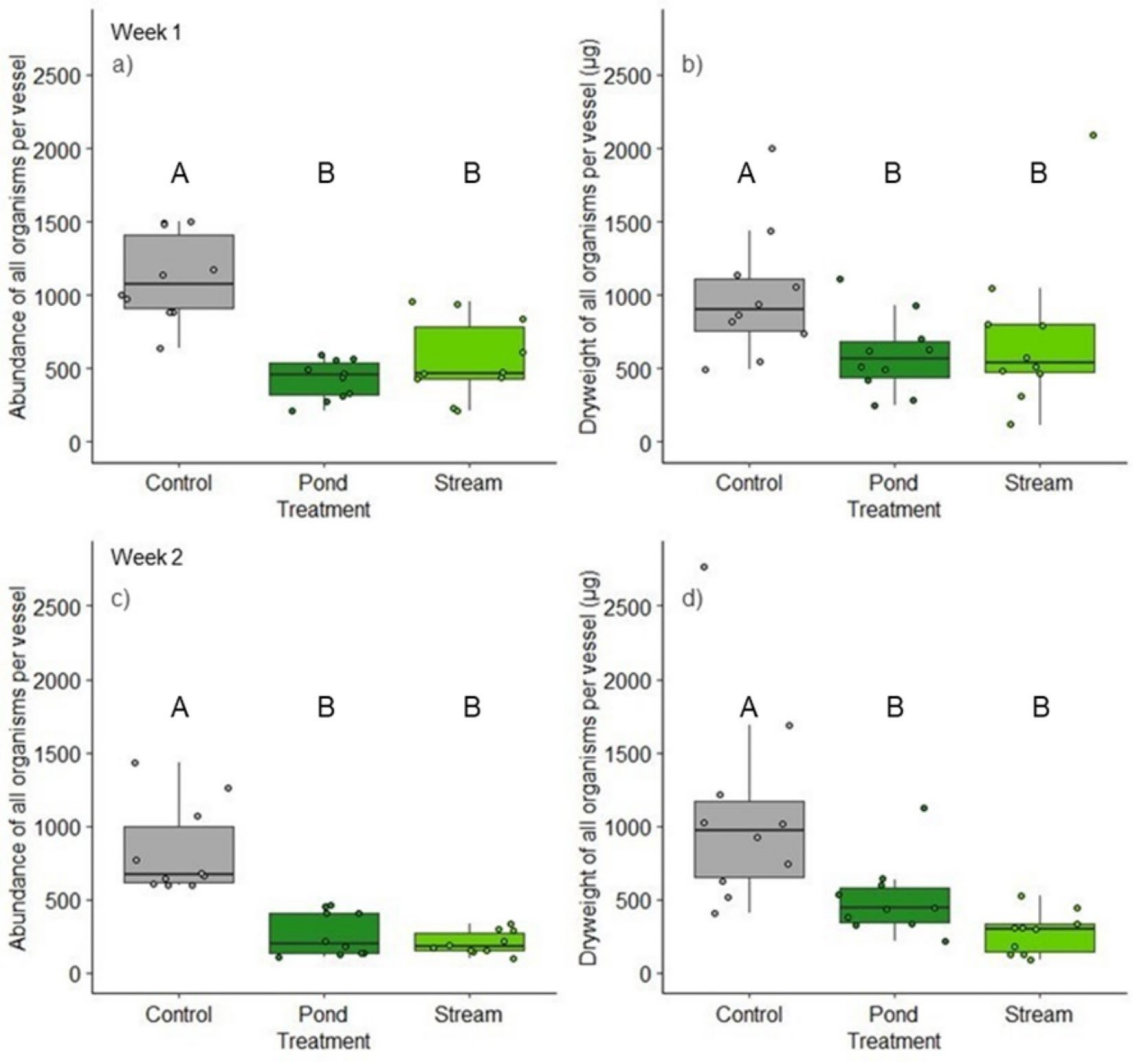
Abundance (left; **a** & **c**) and µg dry weight (right; **b** & **d**) of all benthic organisms per microcosm after week 1 (top; **a** & **b**) and week 2 (bottom; **c** & **d**). Boxplots showing the upper (75%) and lower (25%) quartiles and the median (horizontal line) for the ten replicates (shown as dots) from the controls and the larval treatments with stream or pond larvae. Different letters above the plots indicate significant differences (*p* < 0.05).

**Fig. 3:**
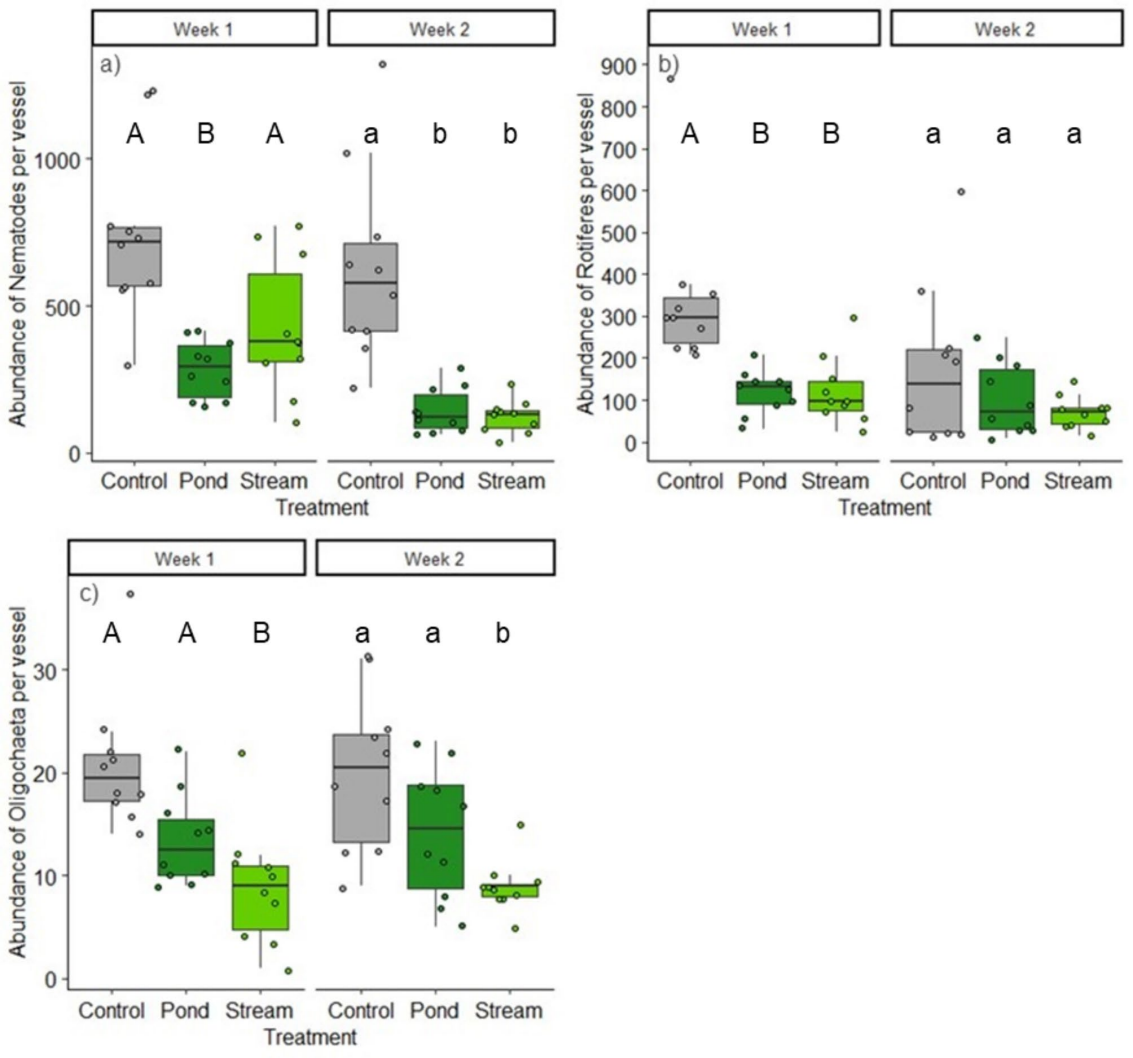
Abundance of nematodes (**a**), rotifers (**b**) and oligochaetes (**c**) per microcosm after week 1 (top) and week 2 (bottom). Boxplots showing the upper (75%) and lower (25%) quartiles and the median (horizontal line) for the ten replicates (shown as dots) from the controls and the treatments with pond or stream larvae. Different letters above the plots indicate significant differences (*p* < 0.05; we tested not between the weeks).

All investigated salamander larvae survived the study and were subsequently returned to their source habitat. After week one, we counted on average 1118 invertebrates (± 298, SD) in the control treatments, while the density was slightly lower at the end of week two (833 ± 309 ind.) (Fig. 2a and c). However, with the exception of rotifers (which were less abundant in week two), we found no significant differences in the abundances of any identified taxon between the controls of week one and week two (Fig. 3b). The total organismal biomass in the controls was 1002.6 µg (± 447 µg, SD) and 1093 µg (± 695 µg, SD) (Fig. 2b and d). Nematodes (65.3% and 73.9%) and rotifers (31.6% and 22.1%) predominated the benthic communities in the controls at both sampling times. Oligochaetes (1.9% and 2.7%) as well as chironomid larvae (< 1% each) represented only a small fraction. In contrast, chironomids (42.2% and 37.2%), oligochaetes (30.8% and 38.3%), and nematodes (25.5% and 20.3%) made up the majority of the organismal biomass at both sampling times, whereas the dry weight of the rotifers was low (< 0.4% at both sampling times). A list of all the collected organisms can be found in the Supplementary Tables S1 & S2.

### Impact of fire salamander on the benthic community

After week one, we observed a significant decrease in the total abundance and biomass of benthic taxa in both larval treatments compared with those in the controls (Fig. 2a and b). The abundance and biomass of benthic species in the treatments with stream larvae decreased significantly between weeks 1 and 2, whereas there was only a slight decrease after week 1 in both parameters in the microcosms with pond larvae (Fig. 2c and d). At the end of the two-week study, the average reduction in abundance was 81.7% (± 6.8%, SD) in microcosms with stream larvae and 76.6% (± 13.4%, SD) in the presents of pond larvae. The total biomass decreased by 72.5% (± 14.3%, SD) and 49.7% (± 25.3%, SD). Overall, we found no significant difference in the total percentage decline in abundance or biomass between the two treatments after two weeks (Tables S1 & S2).

The temporal- and treatment-based effects varied between the taxa found, particularly in the nematodes, oligochaetes and rotifers (Fig. 3). The nematode abundance in the treatments with pond larvae significantly differed from that in the controls (*p* < 0.05), resulting in a mean reduction of 61.4% (± 13.4%, SD) after the first week and a slightly greater reduction of 77.3% (± 12.4%, SD) after week two (Fig. 3a). In the case of nematodes exposed to stream larvae, there was a significant (*p* < 0.05) decrease in abundance only after two weeks (mean reduction of 80.4% ± 13.4%, SD). The abundance of oligochaetes (Fig. 3c) was significantly lower than that in the controls at both sampling times, but only in the treatments with stream larvae. Here, the mean abundance decreased by 57.2% (± 28.5%, SD) and 55.2% (± 12.4%, SD). In contrast, rotifers (Fig. 3b) were similarly affected in both larval treatments. They showed significant lower abundances (*p* < 0.05) after the first week, with a mean reduction of 64.9% (± 23.2%, SD) in the presence of stream larvae and 65.3% (± 15.1%, SD) in microcosms with pond larvae. This effect was no longer detectable at the end of the experiment.

In connection with these abundance reductions, we did not observe any effects on specific size classes, such as a decline in large individuals. All size classes were reduced in proportion to their occurrence. The observed effects on the biomass of the different taxa are largely parallel to the described changes in abundance.

### Larval body measurements and activity

**Fig. 4:**
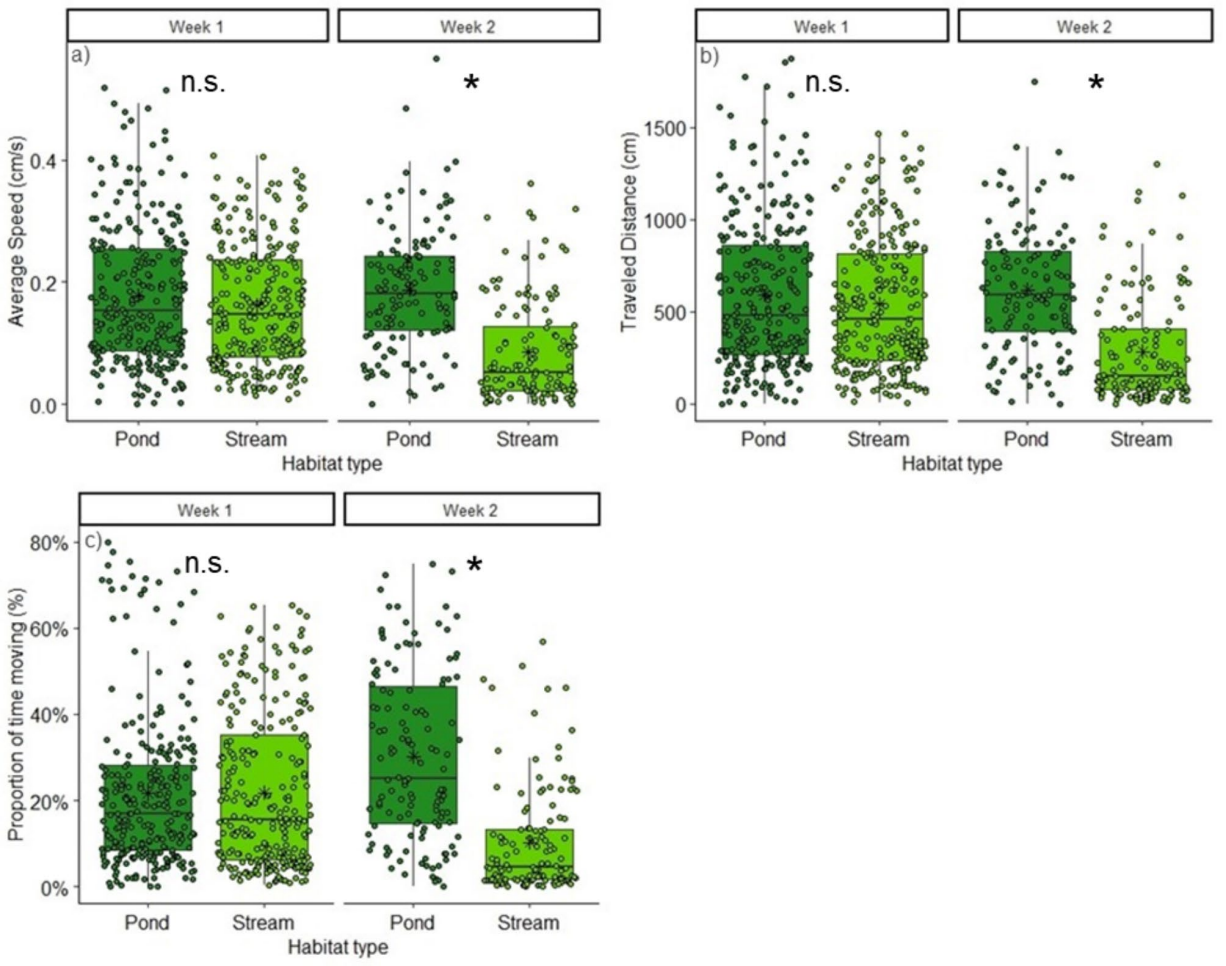
Daytime measurements of the average speed (cm/s) (**a**), distance traveled (cm) (**b**) and proportion of time spent moving (%) (**c**) of the salamander larvae from the stream and pond treatments during the first and second week. Boxplots show the upper (75%) and lower (25%) quartiles and the median (horizontal line), while the dots indicate the 260 (week 1) and 130 (week 2) replicates. n.s. = tested pairs not significant, * = *p* < 0.05.

While the body and gill filament lengths of the stream larvae did not change during the course of the study, the size of the pond larvae increased slightly but significantly (*p* = 0.01) after 2 weeks by an average of 1 mm (± 0.08 mm, SD). Additionally, we observed a significant (*p* = 0.01) reduction in the gill filament length of the pond larvae after just one week. The length of the gill filaments decreased by 1.2 mm (± 0.04 mm, SD). When we analyzed the average speed, travel distance and proportion of time spent by the larvae, we found a significant interaction between the treatments and the sampling time. For the proportion of time moving between the larvae from the two habitat types, we found a significant interaction between the habitat and the week (week 1 or 2) of the experiment (*p* < 0.05). Likewise, we found a significant interaction between the habitat and the week for the travelled distance (*p* < 0.05). We also found a significant interaction for the average speed between the habitat type and the week (*p* < 0.05). All three factors showed consistent values ​​for both larval types after the first week (Fig. 4). However, at the second sampling time, all values were significantly lower (*p* < 0.05) for larvae from streams than for those from their pond equivalents.

## Discussion

Fire salamander larvae can strongly impact meiofaunal and macrofaunal communities. According to our initial assumption (1), in the larval treatments, the abundance and biomass of the benthic taxa were reduced within one week, especially those of nematodes, rotifers and oligochaetes. Another key finding is the different influences of the two larval types. Contrary to our initial assumption (2), the overall effects on abundance and biomass were similar in both larval treatments. However, in treatments with pond larvae, the abundance of meiofaunal nematodes and rotifers was significantly reduced, whereas stream larvae additionally affected oligochaetes. Both findings are in line with our hypotheses 2a and 2b. Our initial expectation (H3) of higher activity among the stream larvae was not confirmed. Even though the salamander activity recorded in the videos does not provide clear evidence of definite feeding behavior, as we filmed them from above, we assume a trophic interaction. The fact that significantly fewer invertebrates were found in the treatments with salamander larvae compared to the controls strongly supports this assumption. Under other scenarios, such as chemical or physical effects (e.g., due to substances released by the larvae or mechanical disturbance of the sediment), considerably more and also strongly decomposed remains of the benthos would have been found. (e.g., body parts of oligochaetes or the cuticle of nematodes). Nevertheless, we discuss these factors below.

After just one week, the abundance of benthos in the 100 cm^2^ test microcosms was reduced by more than 50% by pond and stream larvae, with body sizes ranging from 3 to 3.5 cm. At the end of the 14-day study period, the decrease was greater than 75%. For comparison, Spieth et al.^42^ and Weber & Traunspurger^43^ implemented laboratory experiments with juvenile carps and gudgeons and documented strong effects on the benthic communities. While carps 9–12 cm in body length reduced the abundance by 52% in 2 days, gudgeons (5–7 cm) had a similar effect after 24 h. Both species particularly influenced nematodes and oligochaetes, while in contrast to our study, no effect on rotifers was documented. Even though they are consumed as food in aquaculture^44^, rotifers do not seem to be influenced by fish and macrobenthic taxa in sediment under natural conditions (reviewed by 23,24). Therefore, our results, which show an effect after at least the first 7 days, are surprising. Compared with other benthic organisms, rotifers have a short generation time, react quickly to environmental influences, and therefore generally exhibit strongly fluctuating population dynamics^45–47^. This was also evident in their reduced abundances in the control treatments after 2 weeks. Benthic rotifers and nematodes are typical endobenthic organisms that rarely enter the water column. On the other hand, limnic oligochaetes, for example, from the taxon tubificida, often protrude from the sediment and can serve as prey for visually hunting predators. The individuals we found in the experimental setups mostly had body lengths of more than 3 mm, which was greater than those of the nematodes (mainly < 1.25 mm) or the rotifers (< 0.25 mm). In lotic waters, oligochaetes constitute a significant portion of the food for salamander larvae, alongside various epibenthic organisms such as gammarids^48^. In temporary ponds, oligochaetes are rare, and pelagic taxa like coleopterans, copepods and especially culicids or terrestrial insects from the water surface dominate the larval diet^5^. Hence, stream larvae are likely already habituated to hunt for oligochaetes, which may explain the effect in these treatments. Moreover, salamander larvae can change their foraging strategy through learned behavior^49^, but hunting for prey mostly occurs near the bottom or the water surface (reviewed by 50). Reinhardt et al.^11^ investigated the effects of fire salamander larvae on the invertebrate community in temporal ponds in the field but were unable to find a reduction in common taxa. A likely explanation for this was the importance of terrestrial insects as food sources. However, smaller benthic organisms (nematodes, rotifers, and small oligochaetes) were not considered in this study because of the sampling method used. Generally, the question arises under which abundances of meiofauna, macrofauna, and terrestrial prey in fire salamander larvae a distinct preference for specific prey groups may emerge. Previous studies have shown that fire salamander larvae can adjust their foraging strategy based on the occurrence and behavior of prey taxa but also on environmental factors such as light and water flow^49,51–53^. Although meiofauna may provide a qualitative enhancement to the diet of larger organisms, such as juvenile fish or macroinvertebrates, the existing literature remains insufficient to draw definitive conclusions regarding the ecological significance of a meiofaunal diet^23^. Our results show that, at least under microcosm conditions, there are significant effects on these taxa. Even though larval abundances in streams are lower than those in ponds are, in both habitats, they can reach levels comparable to those in our experimental design^5,11,54,55^. Therefore, a top-down effect on meiofauna is also likely in nature. However, in natural environments, top-down effects may be masked by factors such as rapid recolonization dynamics and reproduction in meiofauna^56,57^ or water drift due to mechanical disturbances^37,58,59^.

The intake of endobenthic organisms requires that the sediment is stirred up and the prey is retained by morphological structures or feeding behavior. The influences of such mechanical sediment reworking and trophic effects are often difficult to separate from each other. Salamander larvae are typically classified as lurking suction feeders^15,60^, but more active hunting strategies are observed in the absence of light^49^. To the best of our knowledge, no studies have documented the process of sediment uptake. Although we were also unable to detect such behavior through overhead camera footage, clear traces of mechanical deposits such as pits were visible. Palmer^61^ and Schatzberger et al.^62^ demonstrated that the mechanical disturbance caused by crabs or fish strongly affects meiofauna. In both studies, nematodes exhibited a particular sensitivity to the modification of the sediment structure. Moreover, the activity of larger organisms can also lead to the transfer of endobenthic organisms into the water column^37^, potentially making them targets for predators or water drift. It has also been demonstrated in larval salamanders that certain macrobenthic taxa are more frequently found in the water column in their presence^63^. In this context, however, the effect of chemical interactions cannot be ruled out. Studies investigating newts have shown that the release of the neurotoxin tetrodotoxin decreases the mobility of benthic macroinvertebrates and affects predator‒prey interactions^64,65^. With respect to fire salamanders, it is generally assumed that toxic alkaloids are mainly restricted to adults. However, these substances have also been detected in prometamorphosis stages and occasionally in young larvae (reviewed by ^66^).

Gut analysis in previous studies clearly revealed that small endobenthic organisms < 1 mm are ingested by salamander larvae^6,7,26^. Since the larvae we examined showed no signs of impending metamorphosis, we assume that their gill rakers had not yet regressed, allowing for the filtration of small prey organisms. The stronger development of gill filaments in the pond larvae may lead to the assumption that the same applies to the remaining gill apparatus. This could explain the more pronounced decrease in nematodes in the first half of the study, when pond larvae were present. The unselective reduction across all size classes also suggests that feeding occurs through filtration. If the larvae were selectively and visually feeding, a stronger effect would have been expected on larger prey organisms. Moreover, the number of oligochaetes in the treatment with pond larvae was not significantly reduced, whereas the stream larvae themselves had a reducing effect on the nematodes from the second week of the study. Thus, our results cannot be explained solely by the morphology of the gill apparatus but rather by specific behavioral traits and preferences. As mentioned above, oligochaetes are a primary food source for stream-dwelling salamander larvae. Therefore, it seems likely that this prey was reduced first before the much smaller and less rewarding nematodes were consumed from the stream larvae. Larvae from temporary ponds, on the other hand, are faced with a significantly lower food supply and rely on consuming all available prey. The consistently high activity of the pond larvae is also in line with such a foraging strategy. The sediment and water used in the study were sourced from a headwater stream and contained no chemical components from predators such as newts, which, in ponds, are a reason for the reduced activity of fire salamander larvae^67^. Unfortunately, our data cannot be used to determine whether activity was lower at night. At the end of the 14-day study period, the salamander larvae in both treatments reduced the biomass of the benthos to a similar extent. However, we only observed length growth in the larvae from the temporary ponds. These larvae may be able to utilize the consumed energy more effectively, which corresponds to their life strategy of starting metamorphosis early before the water dries. Additionally, the gill length decreased, indicating a greater oxygen content in the experimental containers than in the original habitats. Such restructuring of the gill structures within a few days has already been observed by Bond^68^ and Segev et al.^69^ and perhaps allows the larvae to invest the degraded biomass in length growth.

On the basis of the assumption that small endobenthic organisms are not selective and visually hunted but that the gill apparatus plays an important role as a filter organ, further clarification (e.g., film records) of the feeding process is needed. Like bottom-feeding fish, water drawn into the mouth area of salamander larvae is pushed out through the gill slits, while food particles are retained in the gill apparatus. The number and size of gill rakers, and thus the mesh size of this gill net, can vary greatly among different fish species^42^. Additionally, this filtering process is optimized by complex back-flushing of ingested particles, the morphology of the mouth area (e.g., the clamp-like bone structure and the muscular palatal organ) and the presence of numerous taste buds to detect suitable food^70,71^. Chemical food recognition also plays an important role in fire salamander larvae^49^. However, whether the filtering behavior of salamander larvae, with their relatively rigid jaw morphology^15,60^, is as effective as that of fish and is even capable of retaining rotifers has not yet been investigated.

While the dietary spectrum of salamander larvae has already been described by numerous authors, our study shows that such predation can have a significant effect on the benthic community, even on small meiofaunal organisms. Within just a few days, their abundance and biomass were greatly reduced. Due to adaptations to their habitat, this trophic impact differed between larvae from ponds and streams, as did their activity. Overall, larval feeding is still poorly understood, and an important subsequent step is to investigate the results of our microcosm study under field conditions. Such approaches could provide insights into how the results we observe in microcosms manifest under natural conditions and the influence of other factors, such as terrestrial prey (see ^11^), as well as the recolonization or rapid reproduction of prey organisms. Furthermore, for a more precise interpretation of our results, detailed information about the development of gill rakers in fire salamander larvae from ponds or streams is crucial and should be the aim of further studies.

## Electronic supplementary material

Below is the link to the electronic supplementary material.

Supplementary Material 1

## Data Availability

Row data can be found here: https://doi.org/10.4119/unibi/2991035.
