# Supplementary material for: Top-down effects of fire salamander larvae (Salamandra salamandra) on benthic organisms differs between habitat types
Source: Sci Rep. 2025 Apr 16;15:13047. doi: 10.1038/s41598-025-97458-6 (PMC12003822; doi:10.1038/s41598-025-97458-6)
Supplement: Supplementary file 1 — Supplementary Material 1 [file 41598_2025_97458_MOESM1_ESM.doc]

**Table S1:** Mean percentage composition (± SD in the brackets) of organisms in the controls (based on the abundance per microcosm) and the percentage decrease in the treatments with fire salamander larvae from streams and ponds. Rare taxa like ephemeropterans, ceratopogonids, daphnia, mites and tardigrades are not listed.

| Taxon | Week 1 | | | | |  | Week 2 | | | | |
| --- | --- | --- | --- | --- | --- | --- | --- | --- | --- | --- | --- |
|  | Percentage composition controls |  | Pecentage decrease | | |  | Percentage composition controls |  | Pecentage decrease | | |
|  |  |  | Stream |  | Pond |  |  |  | Stream |  | Pond |
|  |  |  |  |  |  |  |  |  |  |  |  |
| Nematoda | 65.3 |  | 42.6 |  | 61.4 |  | 73.9 |  | 80.4 |  | 77.3 |
|  | (± 14.1) |  | (± 31.1) |  | (± 13.4) |  | (± 21.7) |  | (± 9.0) |  | (± 12.4) |
| Rotifera | 31.6 |  | 64.9 |  | 65.3 |  | 22.1 |  | 59.8 |  | 41.1 |
|  | (± 13.8) |  | (± 23.2) |  | (± 15.1) |  | (± 21.6) |  | (± 21.7) |  | (± 49.3) |
| Oligochaeta | 1.9 |  | 57.2 |  | 35.6 |  | 2.7 |  | 55.2 |  | 29.4 |
|  | (± 0.4) |  | (± 28.5) |  | (± 21.5) |  | (± 1.5) |  | (± 12.4) |  | (± 32.1) |
| Copepoda | 0.2 |  | 86.4 |  | 59.1 |  | 0.5 |  | 88.4 |  | 81.4 |
|  | (± 0.2) |  | (± 22.0) |  | (± 33.5) |  | (± 0.2) |  | (± 19.8) |  | (± 36.0) |
| Chironomidae | 0.8 |  | 27.4 |  | 31.0 |  | 0.7 |  | 58.5 |  | 64.2 |
|  | (± 0.4) |  | (± 51.6) |  | (± 38.4) |  | (± 0.5) |  | (± 37.5) |  | (± 13.9) |
| **Total** | **1117.8 Ind.** |  | **49.9** |  | **62.0** |  | **832.9 Ind.** |  | **81.7** |  | **76.5** |
|  | **(± 297.6)** |  | **(± 24.3)** |  | **(± 11.9)** |  | **(± 309.2)** |  | **(± 6.9)** |  | **(± 13.4)** |

**Table S2:** Mean percentage biomass composition (± SD in the brackets) of organisms in the controls and the percentage decrease in the treatments with fire salamander larvae from streams and ponds. Rare taxa like ephemeropterans, ceratopogonids, daphnia, mites and tardigrades are not listed.

| Taxon | Week 1 | | | | |  | Week 2 | | | | |
| --- | --- | --- | --- | --- | --- | --- | --- | --- | --- | --- | --- |
|  | Percentage composition controls |  | Pecentage decrease | | |  | Percentage composition controls |  | Pecentage decrease | | |
|  |  |  | Stream |  | Pond |  |  |  | Stream |  | Pond |
|  |  |  |  |  |  |  |  |  |  |  |  |
| Nematoda | 25.5 |  | 28.9 |  | 58.0 |  | 20.3 |  | 81.5 |  | 75.7 |
|  | (± 9.8) |  | (± 44.5) |  | (± 58.0) |  | (± 9.9) |  | (± 7.5) |  | (± 8.4) |
| Rotifera | 0.4 |  | 64.9 |  | 65.3 |  | 0.3 |  | 79.6 |  | 70.2 |
|  | (± 0.2) |  | (± 23.2) |  | (± 17.2) |  | (± 0.4) |  | (± 11.0) |  | (± 25.0) |
| Oligochaeta | 30.8 |  | 47.3 |  | 40.6 |  | 38.3 |  | 72.5 |  | 49.7 |
|  | (± 8.1) |  | (± 54.7) |  | (± 26.9) |  | (± 15.8) |  | (± 14.3) |  | (± 25.3) |
| Copepoda | 0.9 |  | 65.1 |  | 86.5 |  | 3.8 |  | 93.3 |  | 96.5 |
|  | (± 1.4) |  | (± 99.1) |  | (± 38.1) |  | (± 4.3) |  | (± 14.2) |  | (± 10.5) |
| Chironomidae | 42.2 |  | 11.6 |  | 41.9 |  | 37.2 |  | 71.5 |  | 70.7 |
|  | (± 13.6) |  | (± 67.6) |  | (± 45.0) |  | (± 16.2) |  | (± 27.7) |  | (± 13.2) |
| **Total** | **1002.6 g dry weight** |  | **28.1** |  | **40.6** |  | **1093.0 g dry weight** |  | **72.5** |  | **49.7** |
|  | **(± 447.0)** |  | **(± 54.7)** |  | **(± 26.9)** |  | **(± 695.2)** |  | **(± 14.3)** |  | **(± 25.3)** |
